# Evaluation of Resistance of Oilseed Rape Genotypes to Turnip Yellows Virus

**DOI:** 10.3390/plants12132501

**Published:** 2023-06-30

**Authors:** Emad Ibrahim, Andrea Rychlá, Glenda Alquicer, Lucie Slavíková, Qi Peng, Miroslav Klíma, Viktor Vrbovský, Piotr Trebicki, Jiban Kumar Kundu

**Affiliations:** 1 Crop Research Institute, 16106 Prague, Czech Republic; emad.ibrahim@vurv.cz (E.I.); glenda.alquicer@vurv.cz (G.A.); slavikova@vurv.cz (L.S.); qi.peng@vurv.cz (Q.P.); klima@vurv.cz (M.K.); 2 OSEVA Development and Research Ltd., Oilseed Research Institute, 74601 Opava, Czech Republic; rychla@oseva.cz (A.R.); vrbovsky@oseva.cz (V.V.); 3 Key Laboratory of Cotton and Rapeseed, Ministry of Agriculture, Institute of Industrial Crops, Jiangsu Academy of Agricultural Sciences, Nanjing 210014, China; 4 Applied BioSciences, Macquarie University, Sydney 2109, Australia; piotr.trebicki@mq.edu.au

**Keywords:** aphids, doubled haploids (DH), oilseed rape, resistance, RT-qPCR, TuYV, virus titre

## Abstract

Turnip yellows virus (TuYV), is one of the most important pathogens of oilseed rape, which has caused enormous yield losses in all growing regions of the world in recent years. Therefore, there is a need for resistant varieties for sustainable crop protection. We have investigated the resistance of known varieties and newly developed advanced-breeding lines of oilseed rape to TuYV in greenhouse and field trials. We have analysed the TuYV titre of individual genotypes inoculated with the virus using viruliferous aphids *Myzus persicae*. The genotypes ‘DK Temptation’ and ‘Rescator’ had the lowest and highest virus titres, respectively, and were used as resistant and susceptible models for comparative analyses with other genotypes. In the greenhouse, the best results were obtained with the genotypes ‘OP-8143 DH’ (2.94 × 10^5^ copies), OP-BN-72 (3.29 × 10^5^ copies), ‘Navajo’ (3.58 × 10^5^ copies) and ‘SG-C 21215’ (4.09 × 10^5^ copies), which reached virus titres about 2 times higher than the minimum virus concentration measured in ‘DK Temptation’ (1.80 × 10^5^ copies). In the field trials, the genotypes ‘Navajo’ (3.39 × 10^5^ copies), ‘OP-8148 DH’ (4.44 × 10^5^ copies), ‘SG-C 21215’ (6.80 × 10^5^ copies) and OP-8480 (7.19 × 10^5^ copies) had the lowest virus titres and reached about 3 times the virus titre of DK Temptation (2.54 × 10^5^ copies). Both trials showed that at least two commercial varieties (e.g., DK Temptation, Navajo) and three advanced breeding lines (e.g., OP-8143 DH, OP-BN-72, SG-C 21215) had low titres of the virus after TuYV infection. This indicates a high level of resistance to TuYV in ‘Navajo’ or the newly developed breeding lines and the basis of resistance is probably different from R54 (as in ‘DK Temptation’). Furthermore, the greenhouse trials together with RT -qPCR-based virus titre analysis could be a cost-effective and efficient method to assess the level of resistance of a given genotype to TuYV infection compared to the field trials. However, further research is needed to identify the underlying mechanisms causing this difference in susceptibility.

## 1. Introduction

Oilseed rape (*Brassica napus* L.) is a highly valuable crop grown in many countries and is considered the most important source of vegetable oil in Europe [1,2,3], with a global production of almost 29.2 million tonnes in 2020/2021 (FAO). Plant virus diseases are a major threat to the cultivation and production of oilseed rape. One of the most devastating and economically important viral diseases in oilseed rape is turnip yellows virus (TuYV) [4,5]. TuYV belongs to the genus *Polerovirus* in the family *Solemoviridae* [6] and infects a wide host range, including economically important crops, intercrops, weeds and pasture hosts [7,8,9,10,11]. TuYV causes yellowing, dwarfing, leaf redness, interveinal chlorosis and stunted growth, which can lead to significant yield losses of 11 to 46% in all major oilseed rape growing regions of the world [12,13,14,15,16,17]. TuYV infection is considered one of the main reasons why oilseed rape crops fail to reach their genetic yield potential, as the infection negatively affects all yield components, including the number of plant pods, the number of seeds per pod and the percentage of oil content per seed. Infected plants also have a smaller leaf area and produce fewer primary branches [13].

TuYV is a phloem-limited virus that is transmitted by many different aphid species in a persistent, circulative, and non-propagative manner. The spread of the virus depends on the abundance and movement of the main natural vectors, namely the green peach aphid *Myzus persicae* (Sulzer, 1776), which is known to be the main vector of TuYV with a transmission efficiency of over 90%, and the cabbage aphid *Brevicoryne brassicae* (Linnaeus, 1758), which has been reported to have a lower transmission efficiency [18,19,20,21]. The effective control of the spread and impact of TuYV on yields depend on the flight activities of aphid vectors, the virus reservoir, the plant growth stage and timing of infection, resistant/tolerant varieties and general management strategies (both crop protection and agronomic measures) [12,22]. For example, the higher flight activity of aphids in autumn led to higher infection rates of TuYV in winter oilseed rape crops [12]. Therefore, this virus may become more important for crops due to rising autumn and winter temperatures as a result of global warming, leading to increased vector generation and survival in aro-ecosystems.

In the past, chemical insecticides were widely used to control outbreaks of the virus by targeting the aphid vector. However, studies have shown that reducing aphid numbers does not necessarily reduce the proportion of plants infected with TuYV. Even low numbers of aphids and low numbers of infested plants can lead to high rates of infection with the virus [23]. In addition, overuse of insecticides is neither effective nor sustainable due to the development of aphid resistance to the insecticides or/and environmental safety [22,24]. Moreover, seed treatment with neonicotinoids has been restricted in almost all oilseed rape growing regions in the world, including in the EU (Commission Directive 2010/21/EU). Therefore, there is a growing need for breeding TuYV-resistant genotypes to reduce insecticide use and minimise the impact of TuYV on oilseed rape production. Currently, several sources of host resistance to TuYV are known, such as the lines ‘R54’ introgressed into *B. napus* (‘R54’ is derived from Chinese cabbage, *B. rapa*) and the Korean spring variety *B. napus* var. oleifera ‘Yudal’ [25,26]. A progeny of the R54 line has been used to transfer resistance into commercial varieties such as ‘DK Temptation’ (DSV). ‘Yudal’, on the other hand, is an indigenous inbred line of oilseed rape developed in 1969 as a *B. napus* variety with high erucic acid content and is so far the only line that has shown genetically characterised resistance to TuYV [27,28]. Both sources of resistance are linked to a single dominant QTL on chromosome A04 and provide partial resistance to TuYV [25,29]. In recent years, double haploidy (DH) and marker-assisted selection (MAS) technologies have proven to be valuable tools for breeding for disease resistance in many important crops (reviewed in [30,31]). In oilseed rape, DH populations have been used to develop, evaluate and apply molecular markers for TuYV resistance [29,32]. The impact of new breeding lines resistant to TuYV has not yet led to a sustainable and environmentally sound solution, but this may become increasingly important.

In this study, we analysed virus titre in relation to the resistance of oilseed rape genotypes to TuYV. Seed treatments with neonicotinoids have traditionally been used against the aphids’ vector of the virus. However, almost all oilseed production areas in the world have restricted the use of insecticides for environmental protection. We studied genotypes of different origins, including varieties grown in our regions and advanced-breeding lines developed in our earlier research. We found several genotypes in both the varieties and the breeding lines that had low virus titres both in the greenhouse and in the field trails. Our results provide information on the level of resistance of commercially available varieties, as well as of newly developed breeding lines of oilseed rape, and therefore may contribute to reducing the yield losses caused by the virus infection and finding new sources of resistance for crop improvement.

## 2. Results

### 2.1. Resistance Assessment of Oilseed Rape Genotypes to TuYV Infection Based on Virus Titre by qPCR in Greenhouse and Field Trials

#### 2.1.1. The Greenhouse Trials

**Figure 1:**
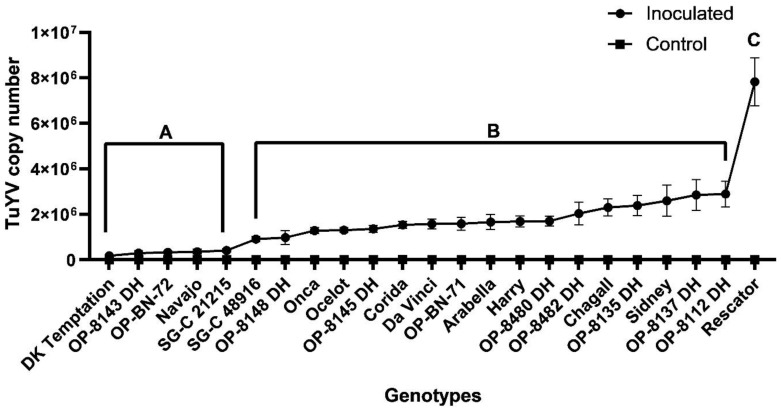
The mean TuYV titre (virus copy number) for each control and inoculated OSR genotypes. Statistically significant differences between the inoculated genotypes and the two reference genotypes. ‘DK Temptation’ as the resistant genotype and ‘Rescator’ as the susceptible genotype based on the average of three-year greenhouse trials. The differences indicated by different letters, A for ‘DK Temptation’, B and C for ‘Rescator’. Bars represent the means and standard errors of 9 biological replicates.

Three individual experiments were conducted to observe the resistance of oilseed rape genotypes associated with TuYV infection based on virus titres (virus copy number) determined 4 weeks after infection using RT-qPCR [11]. The one-way ANOVA revealed a statistically significant difference (F (22, 149) = 16.64), (*p* < 0.0001) in the mean TuYV titre levels between the genotypes. Tukey’s multiple comparisons test revealed a statistically significant difference in the mean TuYV titre between the control and inoculated plants for each genotype (Supplementary Table S1). The titre values are generally negligible or zero in the control plants under the controlled conditions. The same test showed that the highest TuYV titre was found in the susceptible genotype ‘Rescator’ (7.83 × 10^6^ copies), while ‘DK Temptation’ had the lowest virus titre (1.80 × 10^5^ copies) (Supplementary Table S2). Based on the mean value of TuYV titres for each genotype in three greenhouse trials, the genotypes were divided into the following three groups: susceptible, moderate, and highly resistant (Figure 1). Overall, of the 23 genotypes, 4 genotypes, including three advanced breeding lines, namely ‘OP-8143 DH’, ‘OP-BN-72’ and ‘SG-C 21215’, had virus titres about twice the minimum virus concentration measured in ‘DK Temptation’, indicating that these genotypes have similar resistance to TuYV infection as ‘DK Temptation’. Eleven genotypes showed significantly higher titres between 5 and 10 fold of the minimum, indicating moderate resistance to TuYV infection. Finally, seven genotypes developed 10-fold virus titres of the minimum virus titre detected in ‘DK Temptation’, indicating that these genotypes are more susceptible to TuYV infection than ‘DK Temptation’. Interestingly, when comparing the mean TuYV titres of the individual genotypes, we identified several genotypes that consistently had lower virus titres in the three individual greenhouse trials, such as ‘DK Temptation’, ‘OP-8143 DH’, ‘OP-BN-72’, ‘Navajo’ and ‘SG-C 21215’, indicating higher resistance to TuYV infection compared to the other genotypes (Supplementary Figure S1).

#### 2.1.2. The Field Trial

**Figure 2:**
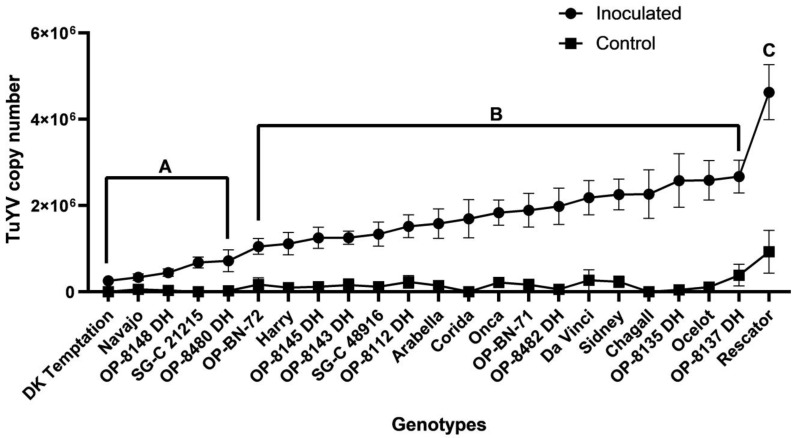
The mean TuYV titres (virus copy number) for each control and inoculated OSR genotypes. Statistically significant differences between the inoculated genotypes and the two reference genotypes. ‘DK Temptation’ as the resistant genotype and ‘Rescator’ as the susceptible genotype based on the average of three-year field trials. The differences indicated by different letters, A for ‘DK Temptation’, B and and C for ‘Rescator’. Bars represent the means and standard errors of 14–16 biological replicates.

Three-year field trials were conducted to screen the resistance levels of 23 oilseed rape genotypes to TuYV infection by measuring the virus titre levels using RT-qPCR. In the second and third year of the study, samples were taken twice a year, the first sample in autumn and the second in early spring. In the first year, the sample was taken only once in early spring. The use of viruliferous aphids *M. persicae* to inoculate TuYV in the experimental plots proved successful in all three years of the study. The one-way ANOVA multiple comparisons test revealed a statistically significant difference (F (22, 310) = 7.546) (*p* < 0.0001) in the mean TuYV titre levels of the genotypes, including both autumn and spring sampling overall during the three-year field trials. The Tukey test for multiple comparisons revealed a statistically significant difference in the mean TuYV titre levels between the control and inoculated plants for each genotype, except for ‘Navajo’ and ‘OP-8480 DH’ (Supplementary Table S3). The titre values of the control plants were taken into account under field conditions in cases of natural infection. The same test also showed that similar results were obtained as in the greenhouse trials. The highest TuYV titre was found in the susceptible genotype ‘Rescator’ (4.63 × 10^6^ copies), while ‘DK Temptation’ had the lowest virus titre (2.54 × 10^5^ copies) (Supplementary Table S4). Similar to the greenhouse trials, out of the 23 genotypes tested, 4 genotypes, including the three advanced breeding lines OP-8148 DH, SG-C 21215, and OP-8480 DH, had virus titres that were not significantly different from the minimum virus concentration detected in ‘DK Temptation’. This suggests that these genotypes have a similar level of resistance to TuYV infection as ‘DK Temptation’. Eight genotypes had virus titres significantly higher (between 4 and 7 times) than the minimum concentration, indicating a moderate level of resistance to TuYV infection. Finally, ten genotypes had virus titres that were more than 7-fold higher than the minimum virus titre detected in ‘DK Temptation’, indicating that these genotypes are more susceptible to TuYV infection (Figure 2). Remarkably, after comparing the mean TuYV titre values of the individual genotypes, we found that some genotypes such as ‘DK Temptation’, ‘Navajo’, ‘OP-8148 DH’, ’SC-C21215’ and ‘OP-8480 DH’ consistently had lower virus titres over the three individual years of the field trial. This result suggests that these genotypes have a higher level of resistance to TuYV infection than the other genotypes (Figure 2, Supplementary Figures S2, S3D and S4D).

#### 2.1.3. The Field Trial (Autumn and Spring Samples 2021/2023)

**Figure 3:**
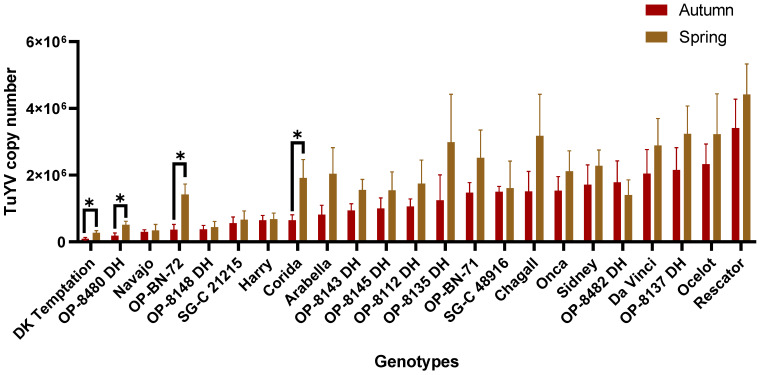
The mean TuYV titres (copy number) for each genotype. Comparison of autumn and spring samples from two-year field trials (years 2021 and 2023). Significant differences are shown after Tukey’s test for multiple comparisons, (* = *p* < 0.05). Bars represent the means and standard errors of six biological replicates.

The results of the two-year field trial (2021/2023) comparing mean TuYV titres (copy number) for each oilseed rape genotype between the autumn (year 2021 + year 2022) and spring (year 2022 + year 2023) samples are shown in Figure 3. The results from the first year of 2020 were not included in this analysis, as sampling was only carried out in the spring season. Analysis with the Tukey test for multiple comparisons again showed that the mean TuYV titre in the autumn samples compared to the spring samples was significantly lower (*p* < 0.05) only for the genotypes ‘DK Temptation’, ‘OP-8480 DH’, ‘OP-BN-72’ and ‘Corida’, yet it remained significantly lower (*p* < 0.05) than the mean titre for the most susceptible genotype in the study (Supplementary Table S5). The greatest difference in the mean TuYV titre between the two seasons was observed for the genotype OP -8135, with the autumn sample having a mean titre of 1.25 × 10^6^ copies and the spring sample a mean titre of 2.99 × 10^6^ copies, almost 2.4 fold. Conversely, the smallest difference in mean TuYV titre was observed in Harry, with a mean titre of 6.48 × 10^5^ copies in the autumn sample and a mean titre of 6.84 × 10^5^ copies in the spring sample. These results show that the virus titre increases in most genotypes from the first infection in autumn to spring. Apparently, the genotypes maintain their resistance levels in the same range within the infection cycles.

#### 2.1.4. The Greenhouse Trial vs. the Field Trial

**Figure 4:**
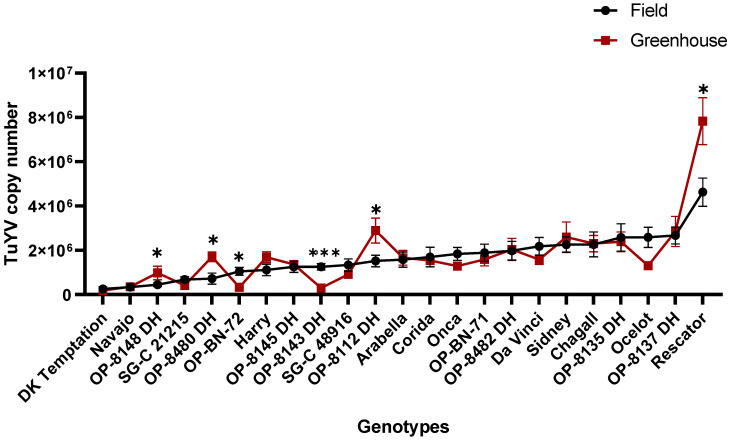
The mean TuYV titres (copy number) for each oilseed rape genotype. Comparison of three-year greenhouse vs. field trials (2020/2023). Significant differences are shown after Tukey’s test for multiple comparisons, (* = *p* < 0.05 and *** = *p* < 0.001,). Bars represent the means and standard errors of 9 biological replicates for the greenhouse trials and 14–16 biological replicates for the field trials.

The comparison of identification results from the greenhouse and field trials conducted over a three-year period (2020–2023) showed a remarkable similarity between the results of the field trials and those of the greenhouse trials in terms of resistant genotypes. Our results revealed that three of the five resistant genotypes (‘DK Temptation’, ‘Navajo’, and ‘SG-C 21215’) were observed together under both conditions. Furthermore, comparison of the results under both conditions showed no significant difference in the mean virus titre of all genotypes tested, except that four genotypes (e.g., ‘OP-8148 DH’, ‘OP-8480 DH’, ‘OP-8112 DH’ and ‘Rescator’) had significantly higher virus concentrations under greenhouse conditions and two genotypes (e.g., ‘OP-BN-72’ and ‘OP-8143 DH’) under field conditions (Figure 4, Supplementary Table S6).

## 3. Discussion

This study provides new information on the resistance or susceptibility of oilseed rape genotypes to turnip yellows virus (TuYV), including both cultivated varieties and newly developed breeding lines, including doubled haploids (DH). Twenty-three genotypes were tested for resistance in the greenhouse and field trials. Both trials showed that at least two commercial varieties (e.g., DK Temptation, Navajo) and three advanced breeding lines (e.g., ‘OP-8143 DH’, ‘OP-BN-72‘, ‘SG-C 21215‘) had low titres of the virus and mild or no symptoms after inoculation of plants with viruliferous aphids *M. persicae*. A low virus titre indicates reduced virus replication, suggesting the high resistance [33] of the genotypes to TuYV. It is noticeable that the variety Temptation, which originated from the resistance line R54 [25], has the highest resistance both in the field and in the greenhouse. ‘DK Temptation’ is an inheritor of the resynthesised *B. napus* line ‘R54’, which confers incomplete resistance to TuYV [25,29]. Two R54-derived varieties ‘Ragnar’ and ‘Violin’ also had showed lower infection rates, suggesting increased resistance to TuYV [34]. Our results showed that TuYV titres in ‘DK Temptation’ were 2–43 times lower than in all other genotypes tested. Therefore, ‘DK Temptation’ was considered a resistant genotype and compared to other genotypes to assess their susceptibility to TuYV infection. A similar resistance spectrum as in the variety ‘DK Temptation’ was also found in the variety ‘Navajo’, where the sources of resistance to TuYV are unknown and which is not based on R54 (see Supplementary Figure S5). The advanced breeding lines tested in our trials were originally developed to achieve a high production value. At the same time, the high resistance of some breeding lines (e.g., ‘OP-8143 DH’, ‘OP-BN-72’, ‘SG-C 21215’) to TuYV increases their value as potential commercial varieties. Although “R54” resistance has been introduced into many commercial varieties, this single source increases selection pressure that may challenge the durability of resistance [35]. Therefore, the variety ‘Navajo’ and the three highly resistant breeding lines described in this study can be used to develop new sources of resistance genes against TuYV.

Accurate quantification methods for measuring virus titres are incredibly important for the resistance screening of plant hosts, as mild or no symptoms may have a higher virus titre or vice-versa [36,37,38]. The relationship between a low virus titre (analysed by qPCR) and the resistance/tolerance of genotypes in viruses infecting cereals is well established [39,40,41,42,43]. Depending on the innate resistance/tolerance or susceptibility of a genotype, a low (or reduced) virus titre correlates with lower virus spread and yield losses [33,44]. In addition to a low virus titre, low infection rates have been associated with resistance to TuYV (BWYV) in a number of varieties (e.g., Tranby, Trigold, Stubby, Banjo, ATR Stingray) [34,45] and accessions (e.g., Liraspa-A, SWU Chinese 3, SWU Chinese 5) in field trials [34]. The low infection rate simultaneously limits the spread of the virus in the crop, and thus reduces crop losses [34]. A large number of commercial varieties with partial resistance to TuYV have been reported from France (e.g., Amalie, Aspire, Annalise, Architect, Ambassador, Artemis, Aurelia, Cadran, Coogan), Germany (e.g., Darling, Dazzler, Ludger, Temptation, Allessandro, Feliciano, Atora, Dominator), Poland (e.g., Addition) and the USA (e.g., DMH440) [26].

We found slight differences in the virus titres of the genotypes between the greenhouse and field trials. In all cases, the TuYV titre is much higher in the greenhouse trials than in the field, which may be due to the more efficient transmission by aphids and virus replication under control conditions. However, the performance of the individual genotypes and their resistance/susceptibility characteristics were more or less maintained in both types of trials (see Figure 4). In addition, the field trials showed that the TuYV titre increased slightly in most genotypes from autumn to spring, but only in a few genotypes, including ‘DK Temptation’, ‘OP-8480 DH’, ‘OP-NB-72’, ‘Corida’, was there a statistically significant difference (Figure 2). The increase in virus titre after winter dormancy could be related to an interruption in the suppression of virus multiplication or development due to rising temperatures during plant development in spring. The increase in virus titre after winter dormancy has been observed in a number of host–virus interactions [46,47,48] and was less pronounced in resistant/tolerant plant genotypes than in susceptible ones [39,49,50]. It is noteworthy that some of the genotypes (e.g., ‘Navajo’, ‘OP-8143 DH’, ‘OP-BN-72’, ‘SG-C 21215’) had low virus titres in both autumn and spring, indicating the stability of high resistance to TuYV in these genotypes. At the same time, the results showed that the genotypes maintained their resistance or susceptibility status both in the initial phase and after dormancy in spring. In field-grown oilseed rape (crops in general), virus titres can vary depending on climatic conditions and the survivability of aphid vectors [51,52,53,54]. The multilayered interaction between plants, aphids and TuYV might have been modulated by additional factors such as temperature, light intensity or day length, so that these factors could have an influence on the course of viral pathogenesis. However, the field tolerance of a genotype is important for yield stability [55], as yield is not affected or only slightly affected by virus infection in a tolerant host [40,56]. Preliminary data show that yield is reduced by up to 20% by TuYV infection (see Supplementary Table S7) and that the variety ‘Navajo’ is one of the most yield-stable genotypes. Similar effects on yield were also observed with the TuYV-resistant variety ‘Caletta’ [29]. However, several years of field data are required for a proper evaluation of the influence of plant resistance on yield. Due to the low virus titre in the variety ‘Navajo’ in both field and greenhouse trials and the high yield stability, this genotype can be used as a parental source for the development of new oilseed resistant varieties or as a reference for the evaluation of resistance to TuYV. Overall, the evaluation of resistance to TuYV in the greenhouse trial described here is an effective and cost-efficient choice for screening genotypes. However, the different stains or isolates of a given virus can cause different levels of resistance in a host genotype depending on the virulence of the virus [39,57]. Recent studies have shown considerable diversity among TuYV isolates [58,59], which should be taken into account when assessing the resistance of genotypes to the virus. Although the use of several distinctly related TuYV isolates could be challenging for genotype resistance assessment, this could lead to a better understanding of resistance to the virus.

## 4. Materials and Methods

### 4.1. Plant Material

The present study was conducted over the last three years (2020–2023) under greenhouse and field conditions to investigate the resistance of 23 *B. napus* genotypes to TuYV. These included 11 commercial varieties (e.g., DK Temptation, Navajo, Harry, Arabella, Onca, Da Vinci, Corida, Sidney, Chagall, Ocelot and Rescator) and 12 advanced breeding lines from the Czech oilseed rape breeding programme (e.g., ‘OP-8112 DH’, ‘OP-8135 DH’, ‘OP-8137 DH’, ‘OP-8143 DH’, ‘OP-8145 DH’, ‘OP-8148 DH’, ‘OP-8480 DH’, ‘OP-8482 DH’, ‘OP-BN-71’, ‘OP-BN-72’, ‘SG-C 21215’, ‘SG-C 48916’) (Table 1). The breeding lines were generated by the crossing of commercial varieties and breeding material with defined qualitative and quantitative parameters according to basic protocols [60,61] with some modifications.

### 4.2. Experimental Plan for Screening Oilseed Rape Genotypes for Resistance to TuYV

**Figure 5:**
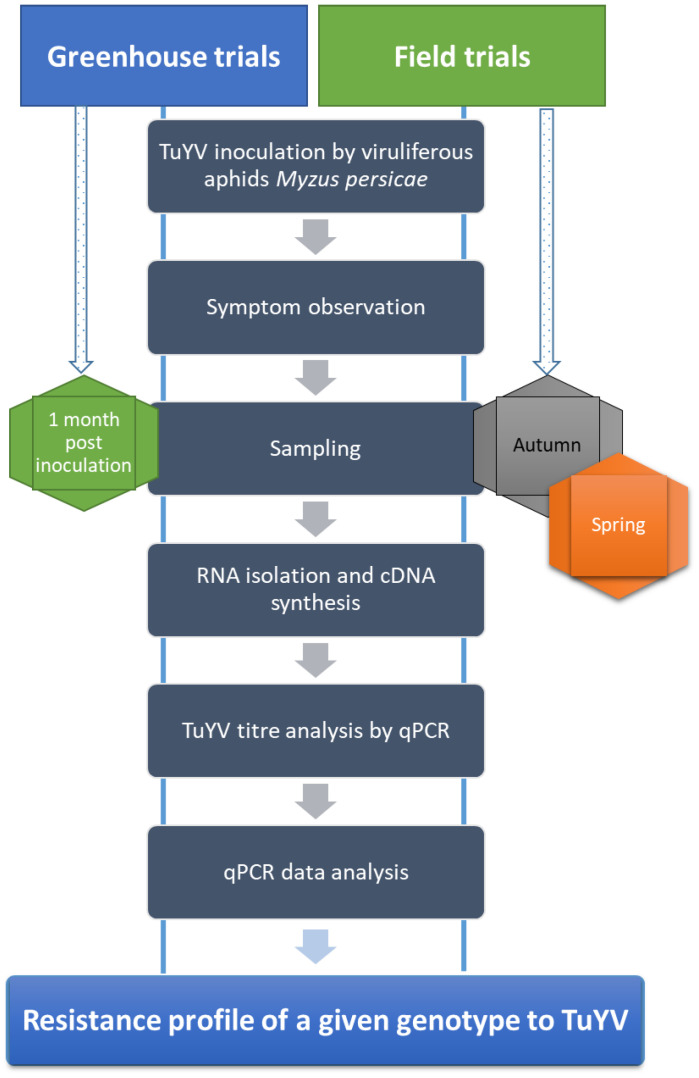
The workflow of assessing the resistance of oilseed rape genotypes to TuYV (an illustration summarising the process of the assessment).

Two types of experiments were conducted for screening the genotypes, including three greenhouse and three field trials. Twenty-three genotypes of different origins, including cultivated varieties and some new breeding lines, were artificially inoculated with the TuYV isolate (accession number OP699027) by the use of viruliferous aphids, *M. persicae*, in both greenhouse and field trials. The virus-free aphids, *M. persicae,* were reared on Chinese cabbage (*Brassica rapa*) in insect-proof cages in the greenhouse. Aphids were collected and allowed, then placed on TuYV-infected *B. napus* plants for three days of the acquisition-access-feeding period (AAFP) (under a 16 h photoperiod at 20 ± 2 °C and 60% humidity). Then, the viruliferous aphids were transferred into the greenhouse and the field trial plants (approximately 5 aphids per individual plant) at the four-leaf stage for three days’ access to the inoculation feeding period (IAFP). For both the greenhouse and field trials, we used the same group of 23 genotypes as non-inoculated control plants as for the TuYV inoculation. In the greenhouse trials, the control plants were kept in insect-proof cages. In the field trials, they were covered with non-woven fabric to isolate them during artificial inoculation. Aphid feeding activity was then inactivated with an insecticide (0.25 mL/L H_2_O acetamiprid). The trials were conducted over three years from 2020 to 2023 for both conditions. The resistance assessment workflow explains the experimental setup in the greenhouse and field trials, which allows a comprehensive evaluation of the resistance of genotypes to TuYV infection under the given conditions (Figure 5).

#### 4.2.1. Greenhouse Experiments

In the greenhouse, the seeds of the genotypes were planted in 10 × 10 cm plastic pots filled with a premixed sterilised substrate, with one plant per pot. The pots were placed in a separate insect-proof cage and were regularly watered and fertilised. The greenhouses had temperatures of 16 °C to 22 °C, a photoperiod of 16/8 h (light/dark) and a humidity of 60%, which is optimal for both plant growth and virus infection. Each experiment consisted of two treatment groups: healthy control plants and inoculated plants. Each genotype was tested in three biological replicates for the control and inoculated plants in each trial, resulting in a total of nine replicates in three trials. Samples from each plant were collected four weeks after inoculation and frozen in liquid nitrogen, grinded and 100 mg aliquots were stored at 80 °C until use for the quantitative PCR analysis.

#### 4.2.2. Field Experiments

Three experiments were conducted in the field, under typical field conditions in small plots representing the control and inoculated plants, with an area of (10 m^2^ per plot) in the experimental fields (of OSEVA Development and Research Ltd. Oilseed Research Institute with a mean temperature of 8.6 °C and mean annual rainfall of 567.6 mm) during 3 growing seasons (2020/2021—1st: 2020 to 2021; 2nd: 2021 to 2022, and 3rd:2022 to 2023). Each genotype was tested in three biological replicates for the control variant (3 non-inoculated plots for each genotype) and the inoculated variant (3 TuYV-inoculated plots for each genotype). Leaf samples of at least fifteen plants were taken at random from each plot (bulk groupings of leaf of plants throughout the plot) at two time points, in autumn and early spring. Samples were frozen in liquid nitrogen, grinded and 100 mg aliquots were stored at 80 °C until use for quantitative PCR analysis.

### 4.3. RNA Isolation and cDNA Preparation

Total RNA was extracted from leaves using a Trizol kit (Thermo Scientific, Wilmington, DE, USA), according to the manufacturer’s protocol. The concentration and purity of the isolated RNA were quantified using the spectrophotometry method (NanoDrop 2000; Thermo Scientific, Wilmington, DE, USA). Complementary DNA was synthesised using a Reverse Transcription System (Promega, Madison, WI, USA). A reaction mixture composed of 1 µg of total RNA, 0.5 µg random hexamer primer, and RevertAid reverse transcriptase 200 U/µL (Thermo Scientific, Waltham, MA, USA) was incubated at 25 °C for 10 min, 42 °C for 1 h, and then the reaction was stopped by heating to 70 °C for 10 min and subsequent chilling on ice following the manufacturer’s protocol.

### 4.4. Analysis of Virus Titre by RT-qPCR

Quantitative analysis of TuYV was performed in each oilseed rape sample using RT-qPCR. RT-qPCR was performed using a LightCycler^®^ 480 (Roche, Basel, Switzerland) with SYBR Green I as described in [36,62]. The LightCycler^®^ 480 Instrument II (Roche, Basel, Switzerland) was used to measure the exact quantity of viral RNA copies present in the cDNA samples using the 384-well plate containing 12 µL of reaction solution per well. The qPCR reaction mixture was composed of 0.5 µL of the TuYVF-K1 and TuYVR-K1 primer pair mix at 10 µM [11], 6 µL of LightCycler^®^ 480 SYBR Green I Master mix with 2× concentrated (Roche, Basel, Switzerland), 4.5 µL of sterile nuclease-free water, and 1 µL of cDNA, resulting in a total volume of 12 µL. The amplification protocol was as follows: denaturation at 95 °C for 10 min, followed by 40 cycles at 95 °C for 5 s, 60 °C for 30 s, and 72 °C for 20 s. Finally, a melting analysis of the PCR products was performed to confirm that only the specific products were amplified. To calculate the TuYVtitre in the tested samples, a ten-fold standard dilution curve was created using the plasmid DNA of a positive TuYV sample as described by Singh and Kundu [63]. The following mathematical formula was used to estimate the number of transcripts: pmol of dsDNA = µg of dsDNA × 10^6^ pg µg −1/660 pmol pg−1/Nb by using Avogadro’s constant (6.023 × 10^23^ molecules mol^−1^) [64]. Three technical replicates of standards and samples were set up. A Ct value of 35 cycles was chosen as the threshold for virus-positive samples; all samples with a Ct value higher than 35 were labelled as negative.

### 4.5. Extraction of Genomic DNA and Detection of Markers by PCR

Genomic DNA was extracted from leaf tissue of 23 *B. napus* genotypes according to the procedure of Edwards et al. [65]. To determine the genotypic resistance of these varieties, PCR amplification of the ‘R54’ TuYV resistance co-segregation markers was performed with specific primers for the markers (R54-1F&R54-1R) and (R54-2F&R54-2R), as previously described by Juergens et al. [29]. The fragment size of the amplification products was used to determine the presence of the marker R54 in the tested oilseed genotypes to establish the relationship between this marker and plant resistance to the virus (Supplementary Table S8).

## 5. Conclusions

TuYV is one of the most important viral pathogens of *B. napus*, causing enormous yield losses worldwide. The virus itself or its Asian strain, brassica yellows virus, is widespread in all growing regions of the world. Seed treatment with neonicotinoids is traditionally used against the aphids’ vector. However, almost all oilseed growing regions in the world have restricted the use of this insecticide for environmental protection. Together with the challenges of global warming, there is a need for sustainable solutions that contribute to the introduction of resistant oilseed varieties. Our results have shown that virus titre analysed by qPCR can effectively define the resistance of genotypes (e.g., DK Temptation, Navajo, OP-8143 DH, OP-BN-72, SG-C 21215) to TuYV. However, additional data on yield reduction in individual genotypes due to TuYV infection could better explain their tolerance to the virus. The greenhouse trial together with RT-qPCR-based virus titre analysis could replace the expensive and laborious field trials to assess the level of resistance of a given genotype to the virus. Nevertheless, a sufficient number of biological and technical replications should be carried out to better assess the genetic resistance of oilseed rape. The development of DH breeding lines in combination with the described methods for resistance evaluation allow effective breeding of *B. napus* for resistance to viruses in general and to TuYV in particular.

## Figures and Tables

**Table 1:** The sources of the tested oilseed rape genotypes.

No.	Name	Original Maintainer (Commercial Varieties) or Origin (Breeding Lines)
1	Arabella *	Semundo Saatzucht GmbH (Germany)
2	Chagall *	Lantmännen ek för (Sweden)
3	Corida *	Selgen, a.s. (Czech Republic)
4	Da Vinci *	SW Seed Hadmersleben GmbH (Germany)
5	DK Temptation *	Deutsche Saatveredelung AG (Germany)
6	Harry *	Saatzucht Donau GmbH & CoKG (Austria)
7	Navajo *	CPB Twyford Ltd., (Great Britain)
8	Ocelot *	Oseva Pro, s.r.o (Czech Republic)
9	Onca *	Oseva Pro, s.r.o (Czech Republic)
10	Rescator *	Selgen, a.s. (Czech Republic)
11	Sidney *	Saatzucht Donau GmbH & CoKG (Austria)
12	OP-8112 DH **	Ornament × MK
13	OP-8135 DH **	Orex × Cortes
14	OP-8137 DH **	Ornament × Lohana
15	OP-8143 DH **	MK × Ladoga
16	OP-8145 DH **	Orex × Cadeli
17	OP-8148 DH *	Orex × Cortes
18	OP-8480 DH **	Hopson × Ladoga
19	OP-8482 DH **	MK × Lohana
20	OP-BN-71 **	(Orion × Sidney) × Cortes
21	OP-BN-72 **	MK × Sidney
22	SG-C 21215 **	Cortes × Vittek
23	SG-C 48916 **	CH111 × Wisent

*—commercial varieties; **—breeding lines; CH111, MK—breeding lines with high oleic acid content; Hopson—white flowering commercial variety; Orex, Ornament—doubled haploid commercial varieties; Cadeli, Cortes, Ladoga, Lohana, Orion, Vittek, Wisent—commercial varieties.

## Data Availability

Data are included in the article or Supplementary Materials.

## Supplementary Materials

The following supporting information can be downloaded at: https://www.mdpi.com/article/10.3390/plants12132501/s1, Figure S1: The mean TuYV titres (copy number) for each oilseed rape genotypes in greenhouse trials; Figure S2: The mean TuYV titres (copy number) for each oilseed rape genotypes from the first year field trial (2020/2021); Figure S3: The mean TuYV titres (copy number) for each oilseed rape genotypes from the second year field trial (2021/2022); Figure S4: The mean TuYV titres (copy number) for each oilseed rape genotypes from the third year field experiment (2022/2023); Figure S5: Molecular marker detection by PCR; Table S1: Significant differences are shown after Tukey’s multiple comparisons; compare the mean TuYV titres of control and inoculated samples of each genotype over three-year greenhouse trials (2020/2023); Table S2: Significant differences are shown after Tukey’s multiple comparisons; compare the mean of each genotype with the mean of the two reference genotypes. ‘DK Temptation’ as the resistant genotype and ‘Rescator’ as the susceptible genotype based on the average of three-year greenhouse trials; Table S3: Significant differences are shown after Tukey’s multiple comparisons; compare the mean TuYV titres of control and inoculated samples of each genotype over three-year field trials (2020/2023); Table S4: Significant differences are shown after Tukey’s multiple comparisons; compare the mean of each genotype with the mean of the two reference genotypes. ‘DK Temptation’ as the resistant genotype and ‘Rescator’ as the susceptible genotype based on the average of three-year field trials; Table S5: Significant differences are shown after Tukey’s test for multiple comparisons. Comparison of autumn and spring samples of the mean TuYV titres (copy number) for each genotype from two-year field trials (2021/2023); Table S6: Significant differences are shown after Tukey’s test for multiple comparisons. Comparison of three year greenhouse trials vs three year field trials (2020-2023); Table S7: Yield reduction of the oilseed rape genotypes due to TuYV infection in 2021/2022; Table S8: Primers used in this study.
