# Supplementary material for: Evaluation of Resistance of Oilseed Rape Genotypes to Turnip Yellows Virus
Source: Plants (Basel). 2023 Jun 30;12(13):2501. doi: 10.3390/plants12132501 (PMC10346663; doi:10.3390/plants12132501)
Supplement: Supplementary file 1 [file plants-12-02501-s001.zip › Supplementary Figures S1-S5.pdf]

Supplementary Figures S1-S5

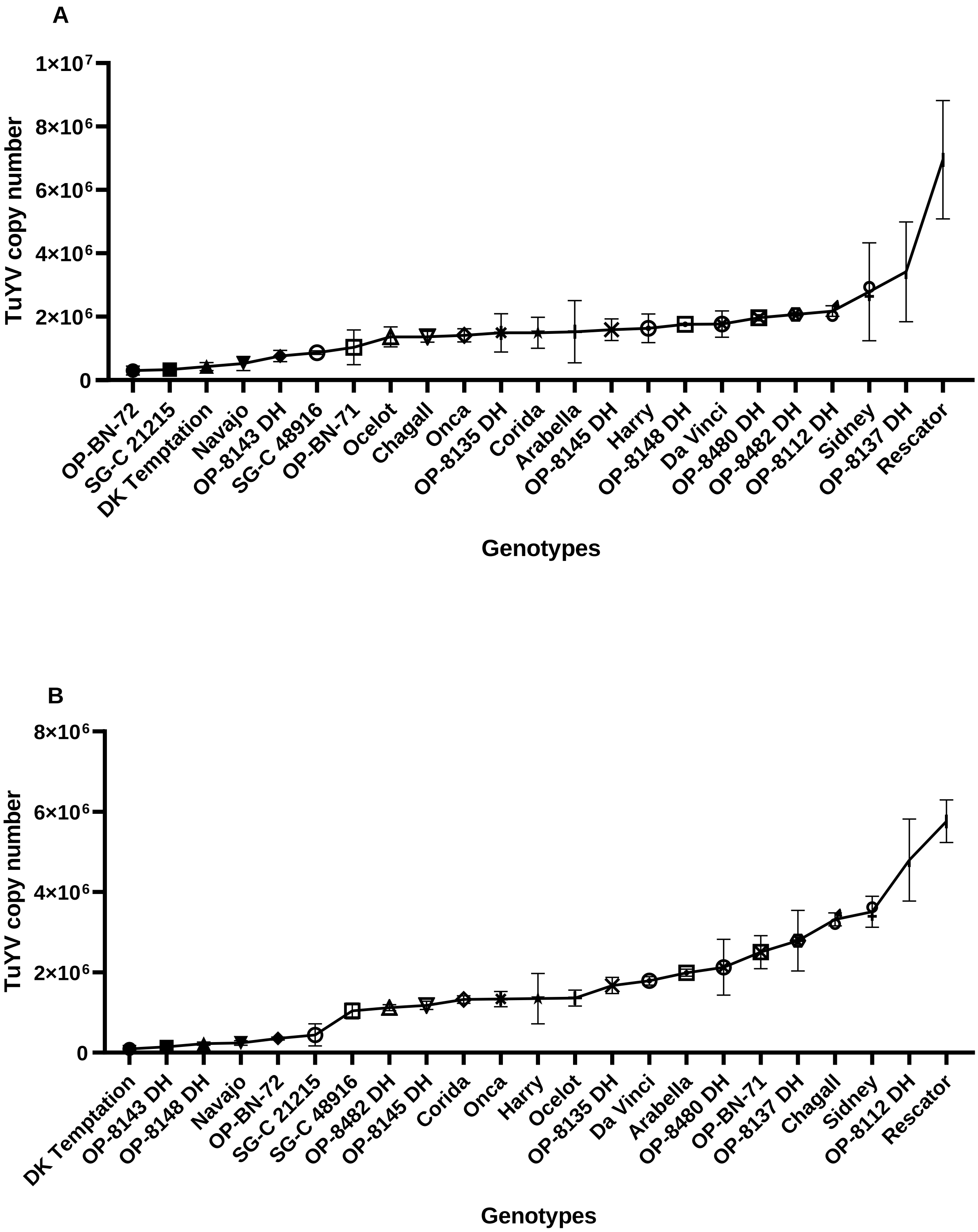

C

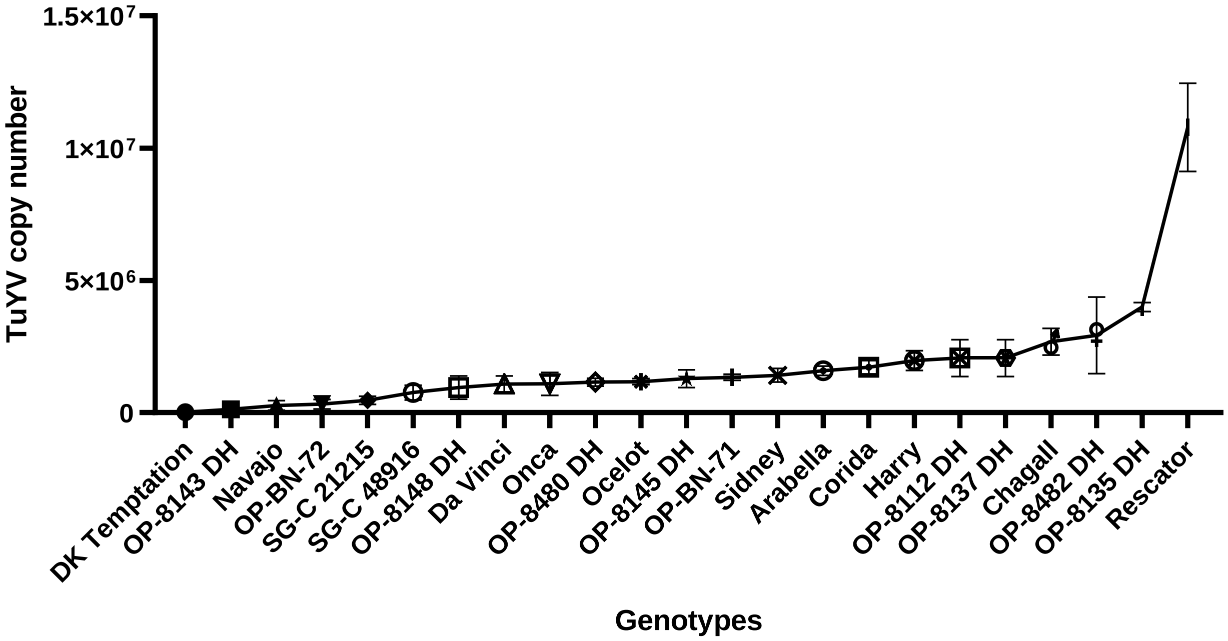

Supplementary Figure S1. The mean TuYV titres (copy number) for each oilseed rape genotypes in the First greenhouse trial (**A**), the second greenhouse trial (**B**) and the third greenhouse trial (**C**). Bars on each column represent standard errors.

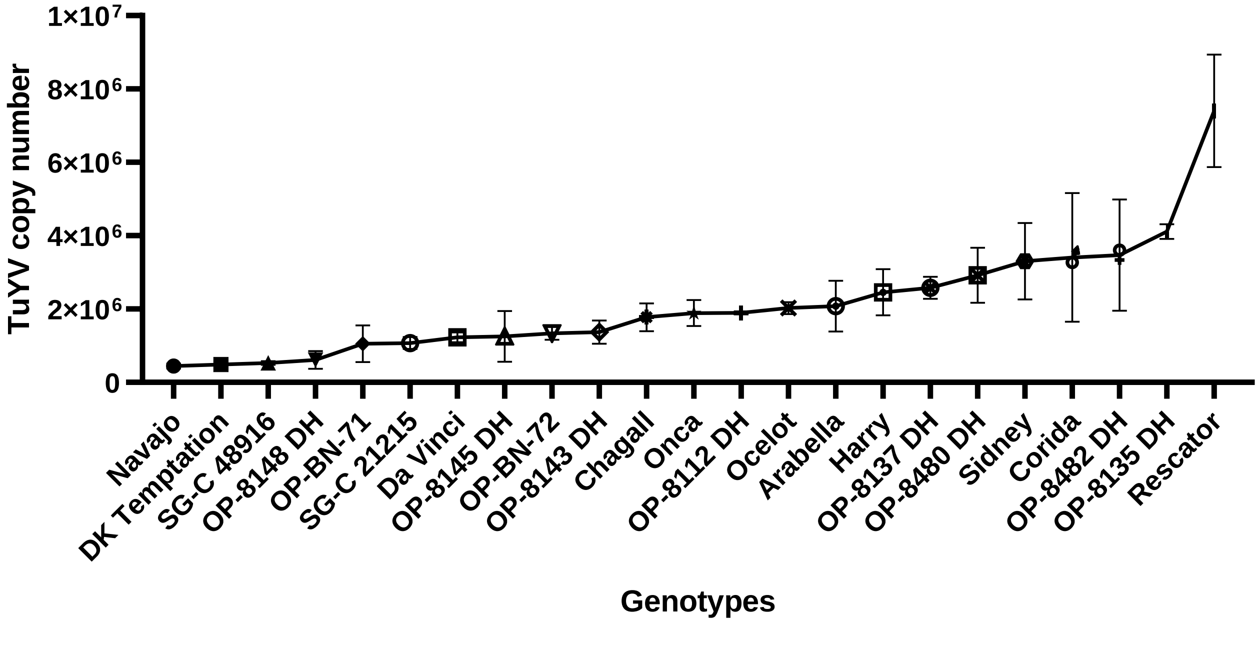

Supplementary Figure S2. The mean TuYV titres (copy number) for each oilseed rape genotypes from the first year field trial (2020/2021)-second sampling in early spring 2021. Bars on each column represent standard errors.

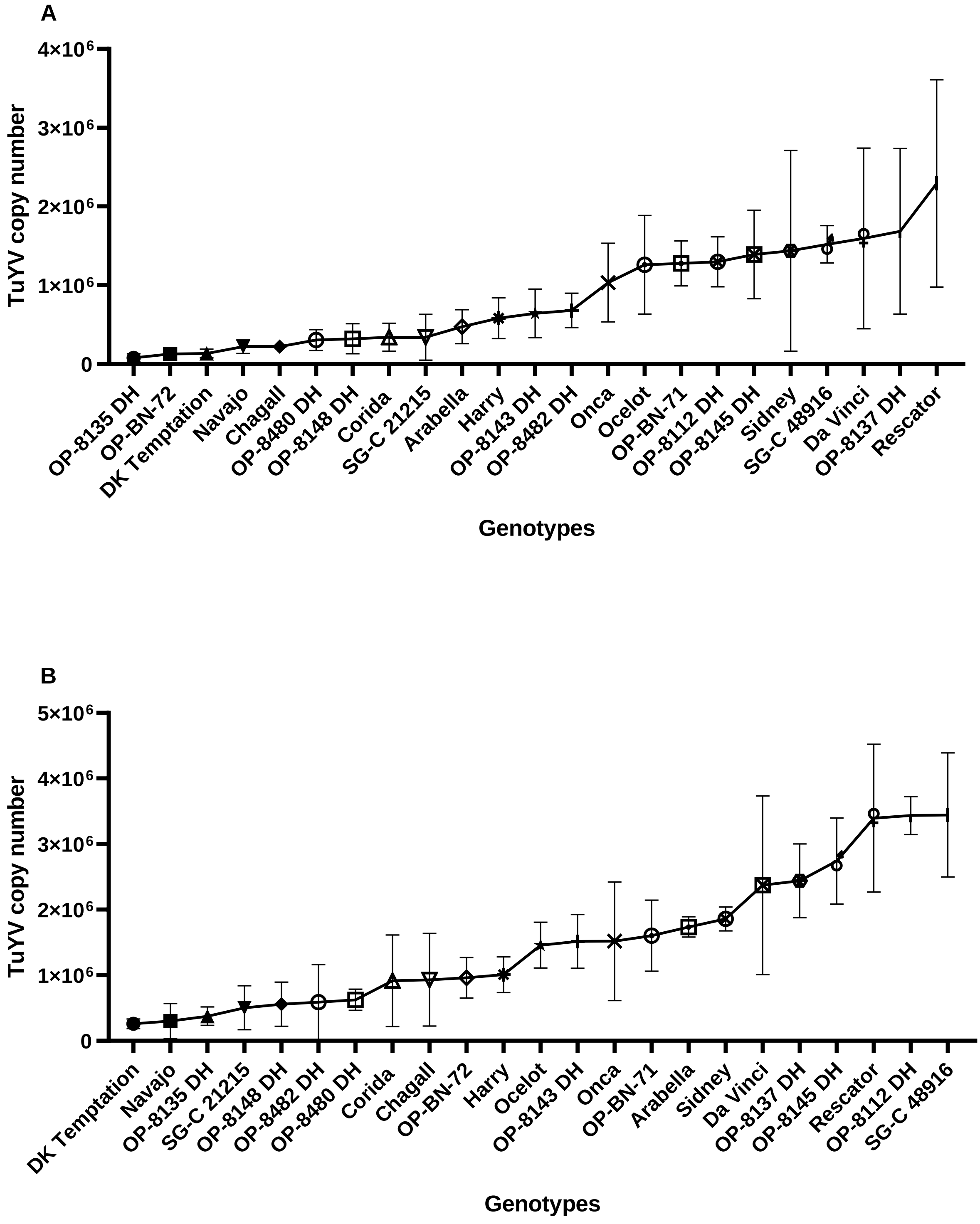
A
TuYV copy number
4×10⁶
3×10⁶
2×10⁶
1×10⁶
0
OP-8135 DH
OP-BN-72
DK Temptation
Navajo
Chagall
OP-8480 DH
OP-8148 DH
Corida
SG-C 21215
Arabella
Harry
OP-8143 DH
OP-8482 DH
Onca
Ocelot
OP-BN-71
OP-8112 DH
OP-8145 DH
Sidney
SG-C 48916
Da Vinci
OP-8137 DH
Rescator
Genotypes
B
TuYV copy number
5×10⁶
4×10⁶
3×10⁶
2×10⁶
1×10⁶
0
DK Temptation
Navajo
OP-8135 DH
SG-C 21215
OP-8148 DH
OP-8482 DH
OP-8480 DH
Corida
Chagall
OP-BN-72
Harry
Ocelot
OP-8143 DH
Onca
OP-BN-71
Arabella
Sidney
Da Vinci
OP-8137 DH
OP-8145 DH
Rescator
OP-8112 DH
SG-C 48916
Genotypes

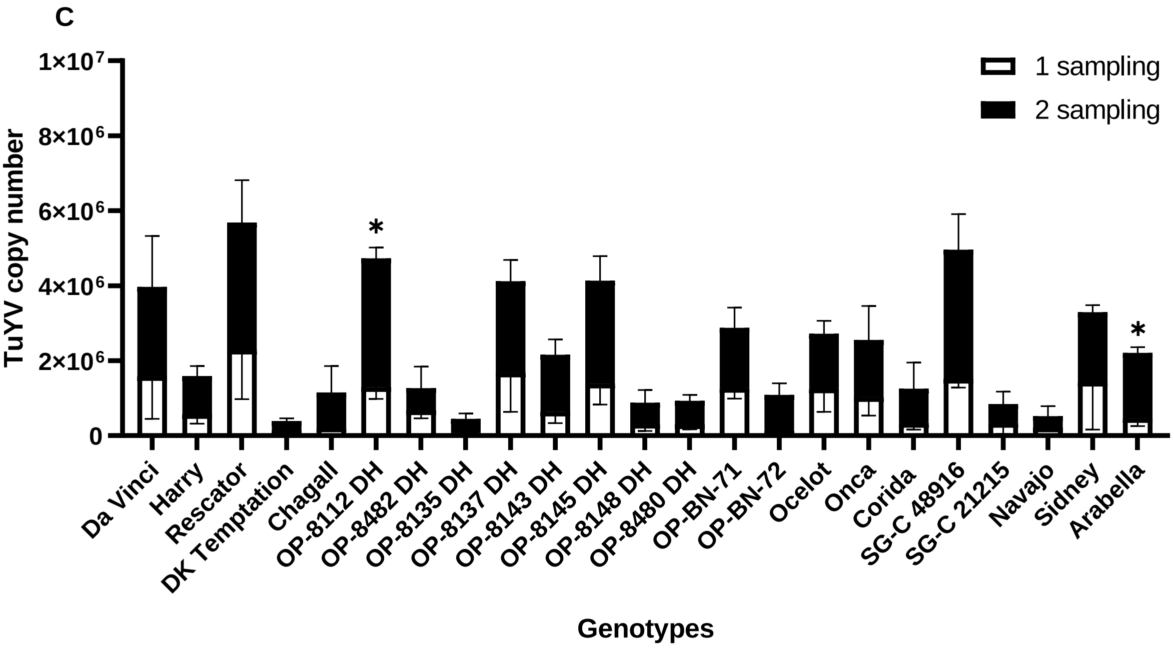

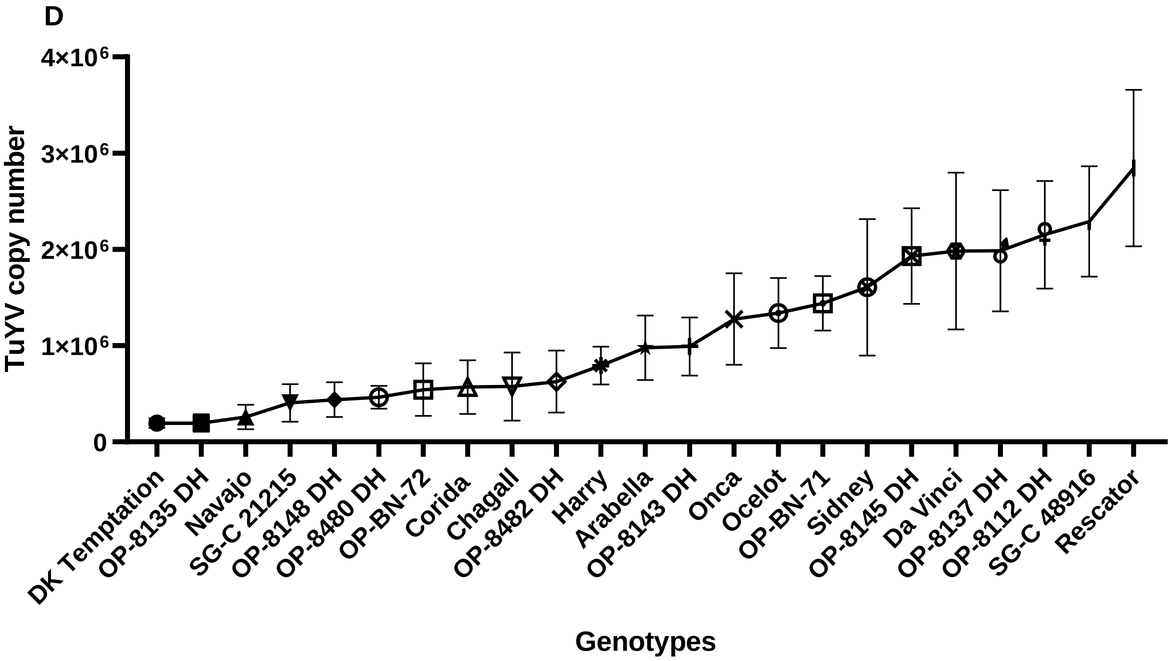

Supplementary Figure S3. The mean TuYV titres (copy number) for each oilseed rape genotypes from the second year field trial (2021/2022). (A) First sampling in autumn 2021, (B) Second sampling in early spring 2022, (C) First sampling in autumn 2021 vs second sampling in early spring 2022, and (D) The total average of both sampling from the second field experiment (2021/2022). Bars on each column represent standard errors.

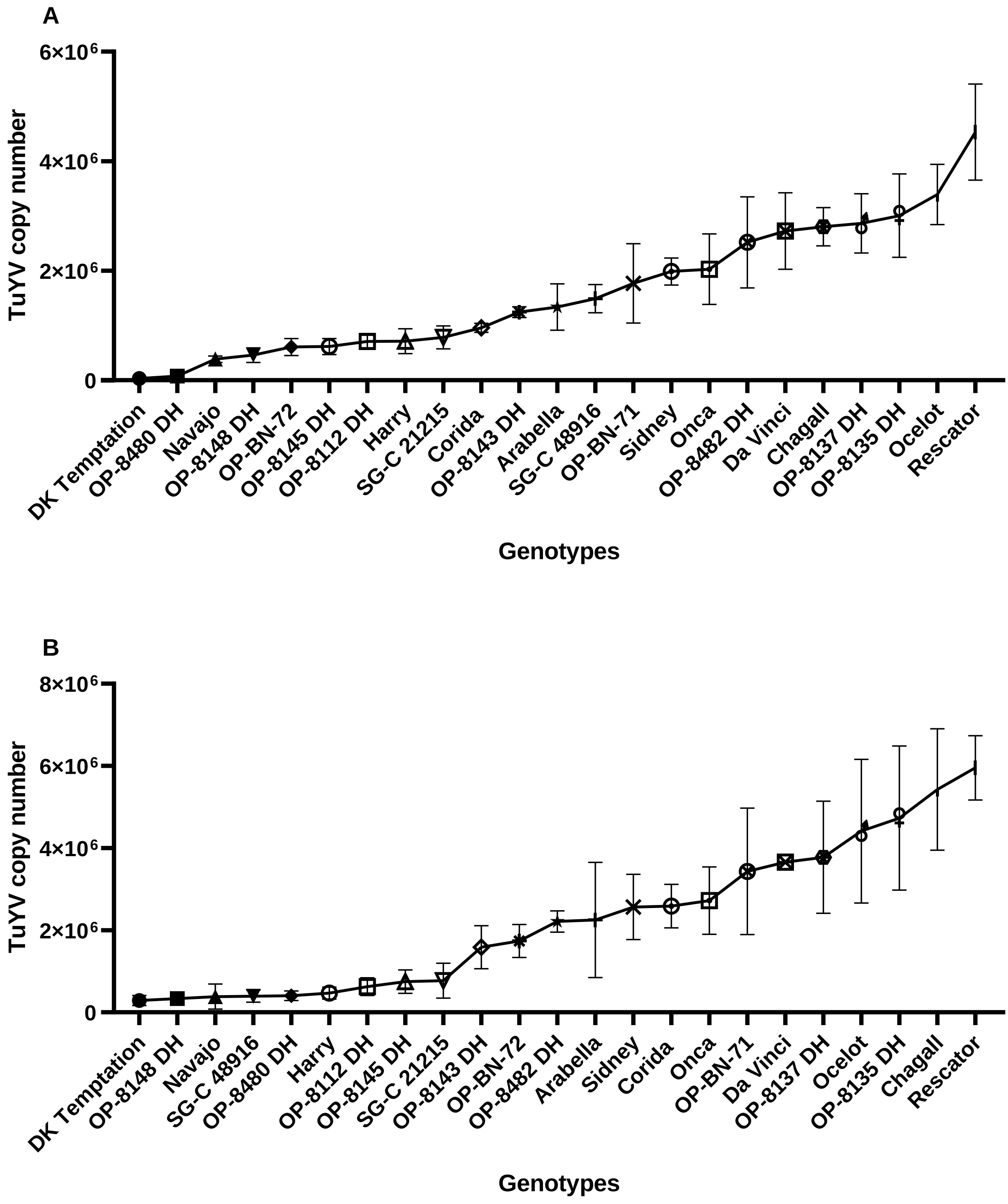
A
TuYV copy number
6×10^6
4×10^6
2×10^6
0
DK Temptation
OP-8480 DH
Navajo
OP-8148 DH
OP-BN-72
OP-8145 DH
OP-8112 DH
Harry
SG-C 21215
Corida
OP-8143 DH
Arabella
SG-C 48916
OP-BN-71
Sidney
Onca
OP-8482 DH
Da Vinci
Chagall
OP-8137 DH
OP-8135 DH
Ocelot
Rescator
Genotypes
B
TuYV copy number
8×10^6
6×10^6
4×10^6
2×10^6
0
DK Temptation
OP-8148 DH
Navajo
SG-C 48916
OP-8480 DH
Harry
OP-8112 DH
OP-8145 DH
SG-C 21215
OP-8143 DH
OP-BN-72
OP-8482 DH
Arabella
Sidney
Corida
Onca
OP-BN-71
Da Vinci
OP-8137 DH
Ocelot
OP-8135 DH
Chagall
Rescator
Genotypes

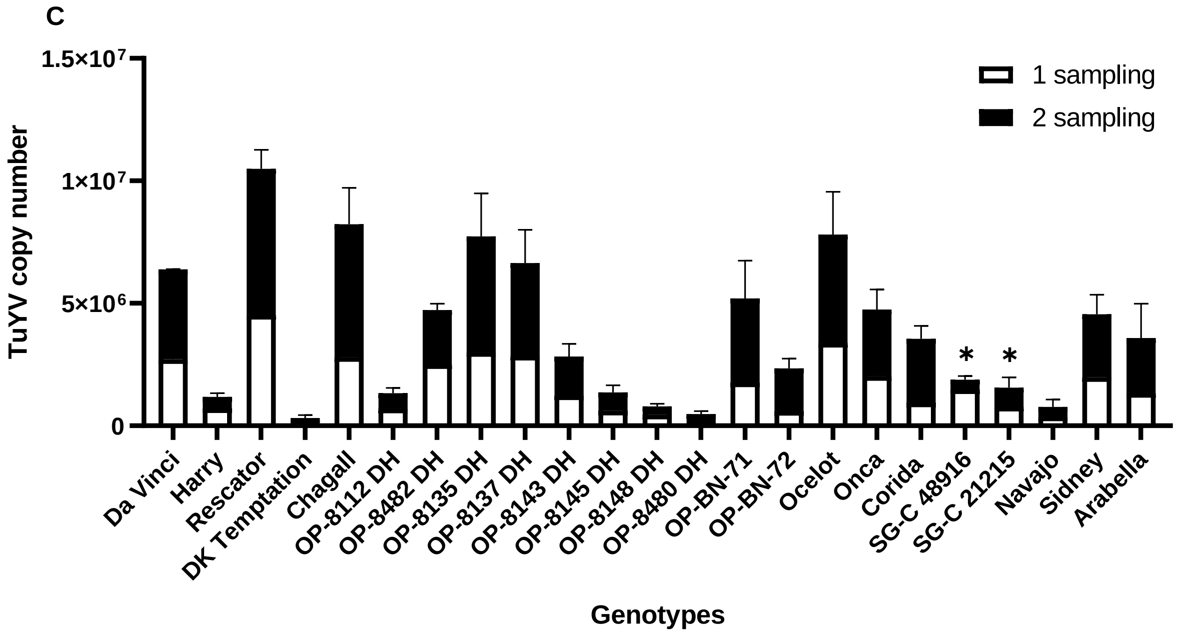

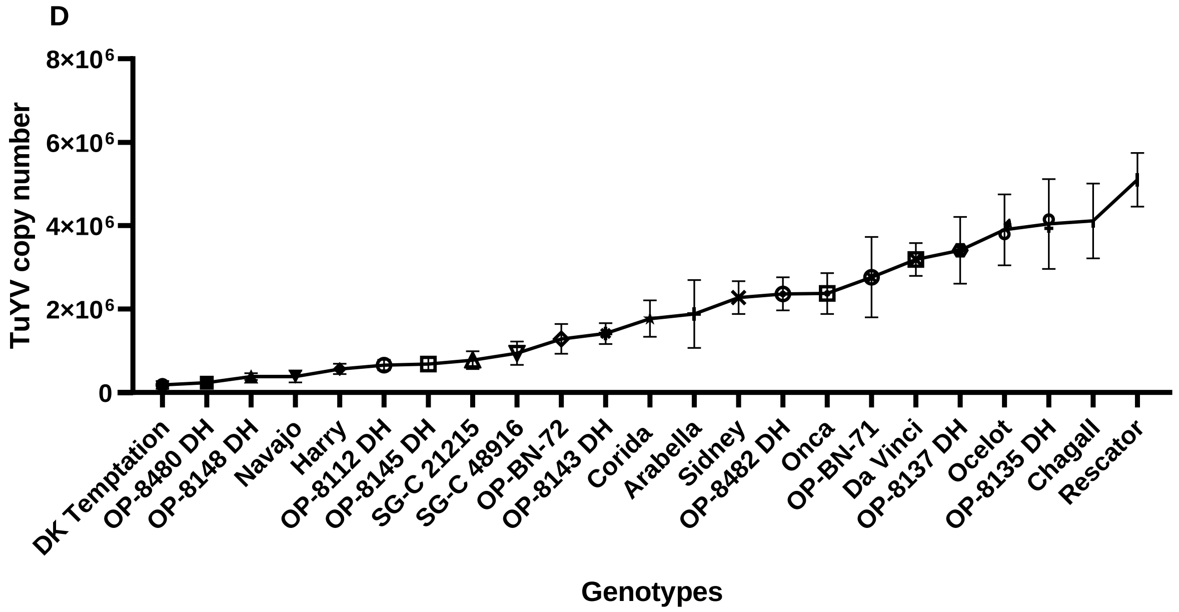

Supplementary Figure S4. The mean TuYV titres (copy number) for each oilseed rape genotypes from the third year field trial (2022/2023). (**A**) First sampling in autumn 2022, (**B**) Second sampling in early spring 2023, (**C**) First sampling in autumn 2022 vs second sampling in early spring 2023, and (**D**) The total average of both sampling from the third field experiment (2022/2023). Bars on each column represent standard errors.

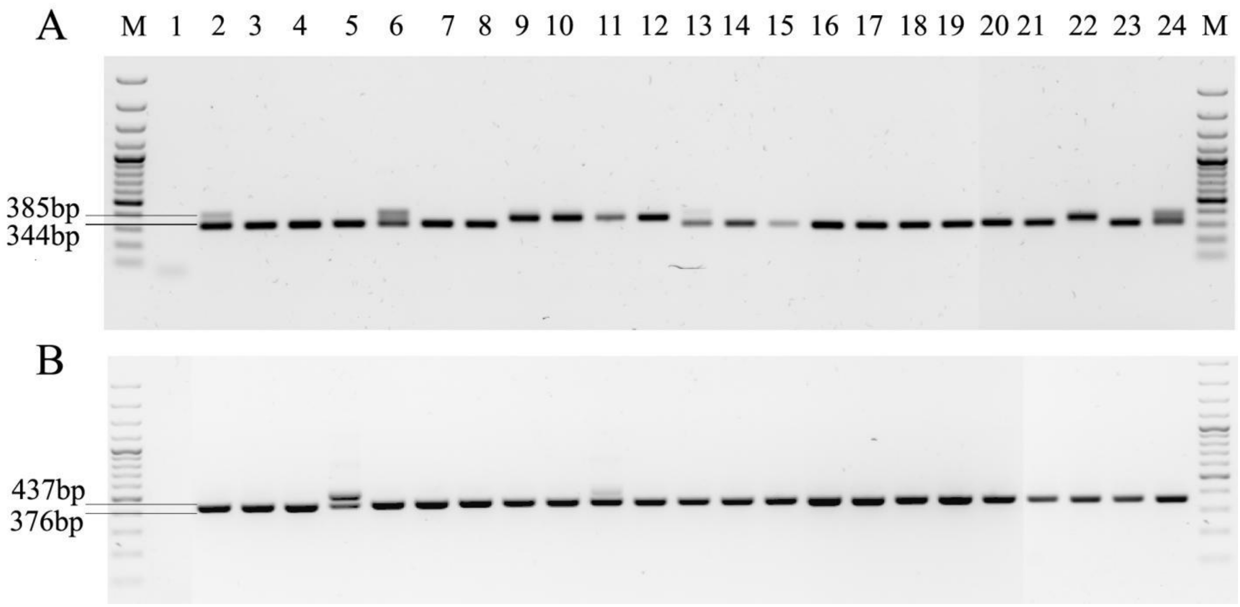

Supplementary Figure S5. Molecular marker detection by PCR using the primer pair R54-1F/R54-1R, Resistant: 344bp, Susceptible: 385bp (lane 1 negative control; lanes 2-24, representing the 23 *B. napus* genotypes: Da Vinci, Harry, Rescator, DK Temptation, Chagall, OP-8112 DH, OP-8284 DH, OP-8135 DH, OP-8137 DH, OP-8143 DH, OP-8145 DH, OP-8148 DH, OP-8480 DH, OP-BN-71, OP-BN-72, Ocelot, Onca, Corida, SG-C-48916, SG-C-21215, Navajo, Sidney, Arabella; lane M GeneRuler 100bp DNA ladder, Thermo fisher Scientific, Wilmington, DE, USA). B. Molecular marker detection by PCR using the primer pair R54-2F/R54-2R, Resistant: 437bp, Susceptible: 376bp (The same as in Figure A).
