# Supplementary material for: Evaluation of Resistance of Oilseed Rape Genotypes to Turnip Yellows Virus
Source: Plants (Basel). 2023 Jun 30;12(13):2501. doi: 10.3390/plants12132501 (PMC10346663; doi:10.3390/plants12132501)
Supplement: Supplementary file 1 [file plants-12-02501-s001.zip › Supplementary Table S1-S8.pdf]

Supplementary Table S1. Significant differences are shown after Tukey's multiple comparisons; compare the mean TuYV titres of control and inoculated samples of each genotype over three-year greenhouse trials (2020/2023). (* = $p < 0.05$, ** = $p < 0.01$, *** = $p < 0.001$, and **** = $p < 0.0001$).

NO.	Genotyes	Summary	P Value
1	DK Temptation	*	0.0443
2	OP-8143 DH	*	0.0371
3	OP-BN-72	**	0.0014
4	Navajo	*	0.0137
5	SG-C 21215	*	0.0133
6	SG-C 48916	***	0.0008
7	OP-8148 DH	*	0.0326
8	Onca	***	0.0004
9	Ocelot	****	<0.0001
10	OP-8145 DH	***	0.0004
11	Corida	***	0.0003
12	Da Vinci	**	0.0016
13	OP-BN-71	**	0.0085
14	Arabella	*	0.0166
15	Harry	**	0.0026
16	OP-8480 DH	**	0.0012
17	OP-8482 DH	*	0.0266
18	Chagall	**	0.0043
19	OP-8135 DH	*	0.0137
20	Sidney	*	0.0417
21	OP-8137 DH	*	0.0285
22	OP-8112 DH	*	0.0351
23	Rescator	**	0.0020

Supplementary Table S2. Significant differences are shown after Tukey's multiple comparisons; compare the mean of each genotype with the mean of the two reference genotypes. 'DK Temptation' as the resistant genotype and 'Rescator' as the susceptible genotype based on the average of three-year greenhouse trials (* = $p < 0.05$, ** = $p < 0.01$, *** = $p < 0.001$, and **** = $p < 0.0001$).

No.	Genotypes	Summary	P value	Genotypes	Summary	P value
1	DK Temptation vs OP-8143 DH	ns	0.3933	Rescator vs DK Temptation	****	<0.0001
2	DK Temptation vs OP-BN-72	ns	0.1557	Rescator vs OP-8143 DH	****	<0.0001
3	DK Temptation vs Navajo	ns	0.1868	Rescator vs OP-BN-72	****	<0.0001
4	DK Temptation vs SG-C 21215	ns	0.0553	Rescator vs Navajo	****	<0.0001
5	DK Temptation vs SG-C 48916	****	<0.0001	Rescator vs SG-C 21215	****	<0.0001
6	DK Temptation vs OP-8148 DH	**	0.0091	Rescator vs SG-C 48916	****	<0.0001
7	DK Temptation vs Onca	****	<0.0001	Rescator vs OP-8148 DH	***	0.0002
8	DK Temptation vs Ocelot	****	<0.0001	Rescator vs Onca	****	<0.0001
9	DK Temptation vs OP-8145 DH	****	<0.0001	Rescator vs Ocelot	****	<0.0001
10	DK Temptation vs Corida	****	<0.0001	Rescator vs OP-8145 DH	****	<0.0001
11	DK Temptation vs Da Vinci	****	<0.0001	Rescator vs Corida	***	0.0001
12	DK Temptation vs OP-BN-71	***	0.0001	Rescator vs Da Vinci	***	0.0002
13	DK Temptation vs Arabella	***	0.0004	Rescator vs OP-BN-71	***	0.0002
14	DK Temptation vs Harry	****	<0.0001	Rescator vs Arabella	****	<0.0001
15	DK Temptation vs OP-8480 DH	****	<0.0001	Rescator vs Harry	****	<0.0001
16	DK Temptation vs OP-8482 DH	***	0.0006	Rescator vs OP-8480 DH	****	<0.0001
17	DK Temptation vs Chagall	****	<0.0001	Rescator vs OP-8482 DH	***	0.001
18	DK Temptation vs OP-8135 DH	***	0.0002	Rescator vs Chagall	***	0.0006
19	DK Temptation vs Sidney	**	0.0012	Rescator vs OP-8135 DH	***	0.0002
20	DK Temptation vs OP-8137 DH	***	0.0005	Rescator vs Sidney	**	0.0016
21	DK Temptation vs OP-8112 DH	****	<0.0001	Rescator vs OP-8137 DH	**	0.0024
22	DK Temptation vs Rescator	****	<0.0001	Rescator vs OP-8112 DH	**	0.002

Supplementary Table S3. Significant differences are shown after Tukey's multiple comparisons; compare the mean TuYV titres of control and inoculated samples of each genotype over three-year field trials (2020/2023). (* = $p < 0.05$, ** = $p < 0.01$, *** = $p < 0.001$, and **** = $p < 0.0001$).

NO.	Genotyes	Summary	P Value
1	DK Temptation	*	0.0182
2	Navajo	No	0.0776
3	OP-8148 DH	*	0.0223
4	SG-C 21215	*	0.0128
5	OP-8480 DH	No	0.1809
6	OP-BN-72	*	0.0219
7	Harry	*	0.0499
8	OP-8145 DH	*	0.0328
9	OP-8143 DH	**	0.0024
10	SG-C 48916	*	0.0414
11	OP-8112 DH	*	0.0225
12	Arabella	*	0.0437
13	Corida	*	0.0492
14	Onca	*	0.0126
15	OP-BN-71	*	0.0355
16	OP-8482 DH	*	0.0339
17	Da Vinci	*	0.0247
18	Sidney	*	0.0107
19	Chagall	*	0.0479
20	OP-8135 DH	*	0.0480
21	Ocelot	*	0.0138
22	OP-8137 DH	**	0.0069
23	Rescator	*	0.0104

Supplementary Table S4. Significant differences are shown after Tukey's multiple comparisons; compare the mean of each genotype with the mean of the two reference genotypes. 'DK Temptation' as the resistant genotype and 'Rescator' as the susceptible genotype based on the average of three-year field trials (* = $p < 0.05$, ** = $p < 0.01$, *** = $p < 0.001$, and **** = $p < 0.0001$).

No.	Genotyes	Summary	P Value	Genotyes	Summary	P Value
1	DK Temptation vs. Navajo	ns	0.3463	Rescator vs. DK Temptation	****	<0.0001
2	DK Temptation vs. OP-8148 DH	ns	0.0582	Rescator vs. Navajo	****	<0.0001
3	DK Temptation vs. SG-C 21215	ns	0.0633	Rescator vs. OP-8148 DH	****	<0.0001
4	DK Temptation vs. OP-8480 DH	ns	0.0803	Rescator vs. SG-C 21215	****	<0.0001
5	DK Temptation vs. OP-BN-72	***	0.0001	Rescator vs. OP-8480 DH	****	<0.0001
6	DK Temptation vs. Harry	**	0.0017	Rescator vs. OP-BN-72	****	<0.0001
7	DK Temptation vs. OP-8145 DH	***	0.0005	Rescator vs. Harry	****	<0.0001
8	DK Temptation vs. OP-8143 DH	****	<0.0001	Rescator vs. OP-8145 DH	****	<0.0001
9	DK Temptation vs. SG-C 48916	***	0.0007	Rescator vs. OP-8143 DH	****	<0.0001
10	DK Temptation vs. OP-8112 DH	****	<0.0001	Rescator vs. SG-C 48916	****	<0.0001
11	DK Temptation vs. Arabella	***	0.0005	Rescator vs. OP-8112 DH	****	<0.0001
12	DK Temptation vs. Corida	**	0.0031	Rescator vs. Arabella	****	<0.0001
13	DK Temptation vs. Onca	****	<0.0001	Rescator vs. Corida	****	<0.0001
14	DK Temptation vs. OP-BN-71	***	0.0002	Rescator vs. Onca	****	<0.0001
15	DK Temptation vs. OP-8482 DH	***	0.0003	Rescator vs. OP-BN-71	****	<0.0001
16	DK Temptation vs. Da Vinci	****	<0.0001	Rescator vs. OP-8482 DH	****	<0.0001
17	DK Temptation vs. Sidney	****	<0.0001	Rescator vs. Da Vinci	****	<0.0001
18	DK Temptation vs. Chagall	**	0.0013	Rescator vs. Sidney	****	<0.0001
19	DK Temptation vs. OP-8135 DH	***	0.0006	Rescator vs. Chagall	****	<0.0001
20	DK Temptation vs. Ocelot	****	<0.0001	Rescator vs. OP-8135 DH	**	0.0014
21	DK Temptation vs. OP-8137 DH	****	<0.0001	Rescator vs. Ocelot	**	0.0011
22	DK Temptation vs. Rescator	****	<0.0001	Rescator vs. OP-8137 DH	**	0.0029

Supplementary Table S5. Significant differences are shown after Tukey's test for multiple comparisons. Comparison of autumn and spring samples of the mean TuYV titres (copy number) for each genotype from two-year field trials (2021/2023). (* = $p < 0.05$, ** = $p < 0.01$, *** = $p < 0.001$, and **** = $p < 0.0001$).

No.	Genotyes	Summary	P Value
1	Da Vinci	ns	0.461075
2	Harry	ns	0.884075
3	Rescator	ns	0.446187
4	DK Temptation	*	0.048864
5	Chagall	ns	0.255236
6	OP-8112 DH	ns	0.379159
7	OP-8482 DH	ns	0.632211
8	OP-8135 DH	ns	0.315565
9	OP-8137 DH	ns	0.34049
10	OP-8143 DH	ns	0.121665
11	OP-8145 DH	ns	0.394665
12	OP-8148 DH	*	0.750193
13	OP-8480 DH	ns	0.031134
14	OP-BN-71	ns	0.30746
15	OP-BN-72	*	0.025303
16	Ocelot	ns	0.497651
17	Onca	ns	0.44745
18	Corida	*	0.039289
19	SG-C 48916	ns	0.887754
20	SG-C 21215	ns	0.752042
21	Navajo	ns	0.844212
22	Sidney	ns	0.484067
23	Arabella	ns	0.177339

Supplementary Table S6. Significant differences are shown after Tukey's test for multiple comparisons. Comparison of three year greenhouse trials vs three year field trials (2020-2023). (* = $p < 0.05$, ** = $p < 0.01$, *** = $p < 0.001$, and **** = $p < 0.0001$).

No.	Genotyes	Summary	P Value
1	DK Temptation	ns	0.396262
2	Navajo	ns	0.883450
3	OP-8148 DH	*	0.033463
4	SG-C 21215	ns	0.144665
5	OP-8480 DH	*	0.017391
6	OP-BN-72	*	0.010909
7	Harry	ns	0.150106
8	OP-8145 DH	ns	0.764876
9	OP-8143 DH	***	0.000321
10	SG-C 48916	ns	0.323163
11	OP-8112 DH	*	0.020351
12	Arabella	ns	0.874104
13	Corida	ns	0.819358
14	Onca	ns	0.230112
15	OP-BN-71	ns	0.613403
16	OP-8482 DH	ns	0.938538
17	Da Vinci	ns	0.319294
18	Sidney	ns	0.626169
19	Chagall	ns	0.967130
20	OP-8135 DH	ns	0.823471
21	Ocelot	ns	0.075176
22	OP-8137 DH	ns	0.804402
23	Rescator	*	0.011231

Supplementary Table S7. Yield reduction of the oilseed rape genotypes due to TuYV infection in 2021/2022.

	**Non inoculated control plots**					**TuYV inoculated plots**						**% of yield reduction due to TuYV infection**
Genotype	Plot number	Yield/ ha	moisture	recalculated yield/ha	Average yield/ha	Genotype	Plot number	Yield/ha	moisture	recalculated yield/ha	Average yield/ha	
Da Vinci	**661**	4.55	7.9	**4.72**	5.24	Da Vinci	**662**	3.9	8.2	**4.04**	4.66	**89.01**
	**744**	5.85	8.2	**6.06**			**743**	4.9	8.2	**5.07**		
	**775**	4.65	5.6	**4.93**			**776**	4.7	8	**4.87**		
Harry	**663**	5.15	8.2	**5.33**	5.65	Harry	**664**	4.7	7.1	**4.92**	4.93	**87.33**
	**730**	5.5	7.9	**5.71**			**729**	4.75	7.7	**4.94**		
	**807**	5.75	9	**5.91**			**808**	4.8	8.7	**4.95**		
Rescator	**665**	5.5	7.7	**5.72**	5.45	Rescator	**666**	4.25	7.6	**4.42**	4.90	**89.79**
	**708**	4.8	7.5	**5.00**			**707**	4.95	7.3	**5.17**		
	**799**	5.45	8.2	**5.64**			**800**	4.95	8.7	**5.10**		
	**667**	6.1	7.8	**6.34**	6.16		**668**	4.45	7.3	**4.64**	5.23	**85.03**

DK Temptation	**732**	6.05	8.3	**6.26**		DK Temptation	**731**	4.95	7.8	**5.14**		
	**781**	5.75	9.6	**5.88**			**782**	5.7	7.9	**5.92**		
Chagall	**669**	5.25	7.5	**5.47**	5.21	Chagall	**670**	4.45	7.7	**4.63**	4.65	**89.33**
	**710**	4.5	8.1	**4.66**			**709**	4.65	8	**4.82**		
	**791**	5.3	8.1	**5.49**			**792**	4.35	8.1	**4.51**		
OP-8112 DH	**671**	5.7	8.3	**5.89**	5.37	OP-8112 DH	**672**	4.65	7.3	**4.85**	5.29	**98.50**
	**718**	5.05	9.6	**5.16**			**717**	4.85	8.2	**5.02**		
	**801**	4.9	8.6	**5.05**			**802**	5.8	8.4	**5.99**		
7 OP-8482 DH	**673**	5.6	7.9	**5.81**	5.37	7 OP-8482 DH	**674**	4.7	8	**4.87**	4.91	**91.47**
	**724**	4.7	8.1	**4.87**			**723**	4.75	8.3	**4.91**		
	**773**	5.25	8.3	**5.43**			**774**	4.8	8.6	**4.95**		
OP-8135 DH	**675**	5.55	8.4	**5.73**	5.77	OP-8135 DH	**676**	4.1	7.7	**4.26**	4.66	**80.88**
	**740**	5.4	8	**5.60**			**739**	4.95	8.4	**5.11**		

	**789**	5.7	7	**5.97**			**790**	4.45	8	**4.61**		
OP-8137 DH	**677**	5	8.2	**5.18**	4.92	OP-8137 DH	**678**	4.2	8.2	**4.35**	4.61	**93.72**
	**752**	4.65	8.2	**4.81**			**751**	4.7	8.4	**4.86**		
	**813**	4.65	9.2	**4.77**			**814**	4.5	8.9	**4.63**		
OP-8143 DH	**679**	5.6	8.1	**5.80**	5.20	OP-8143 DH	**680**	3.95	7.9	**4.10**	4.45	**85.51**
	**716**	4.8	8.7	**4.95**			**715**	4.05	9	**4.16**		
	**817**	4.7	8.5	**4.85**			**818**	4.9	8.1	**5.08**		
OP-8145 DH	**681**	6	8.7	**6.18**	6.11	OP-8145 DH	**682**	4.55	8.4	**4.70**	4.95	**81.06**
	**734**	6.2	9.3	**6.35**			**733**	4.9	8.4	**5.06**		
	**805**	5.6	8.3	**5.79**			**806**	4.95	8.9	**5.09**		
OP-8148 DH	**683**	4.9	8.6	**5.05**	5.20	OP-8148 DH	**684**	4.45	7.8	**4.62**	4.55	**87.60**
	**738**	5.35	8.3	**5.53**			**737**	4.2	8.2	**4.35**		
	**797**	4.85	8.5	**5.01**			**798**	4.55	8.7	**4.69**		

OP-8480 DH	**685**	3.75	7.9	**3.89**	4.14	OP-8480 DH	**686**	3.15	8.6	**3.25**	3.35	**80.98**
	**722**	3.55	8.6	**3.66**			**721**	3.65	7.6	**3.80**		
	**777**	4.7	8.3	**4.86**			**778**	2.9	8.1	**3.00**		
OP-BN-71	**687**	4.75	8.2	**4.92**	5.73	OP-BN-71	**688**	4.95	7.4	**5.16**	5.12	**89.33**
	**736**	6.05	8.1	**6.27**			**735**	5	8	**5.19**		
	**783**	5.8	8.3	**6.00**			**784**	4.85	8.6	**5.00**		
OP-BN-72	**689**	4	8.5	**4.13**	5.21	OP-BN-72	**690**	3.85	8.4	**3.98**	4.61	**88.39**
	**750**	6.15	8	**6.38**			**749**	4.95	8.3	**5.12**		
	**787**	5	9.1	**5.13**			**788**	4.6	9	**4.73**		
Ocelot	**691**	5.25	8.7	**5.41**	5.75	Ocelot	**692**	4.85	7.9	**5.03**	5.05	**87.75**
	**742**	6.1	8.7	**6.29**			**741**	5.15	9	**5.29**		
	**815**	5.4	8.7	**5.56**			**816**	4.65	8.1	**4.82**		
Onca	**693**	4.15	7.9	**4.31**	5.39	Onca	**694**	3.95	8.2	**4.09**	4.46	**82.91**

	**728**	5.65	7.9	**5.86**			**727**	4.85	8	**5.03**		
	**785**	5.7	6.7	**5.98**			**786**	4.15	8.7	**4.28**		
Corida	**695**	5.2	9	**5.34**	5.43	Corida	**696**	4.75	8	**4.93**	5.19	**95.61**
	**748**	5.75	7.5	**5.99**			**747**	5.05	7.8	**5.25**		
	**811**	4.8	8.3	**4.96**			**812**	5.25	8.7	**5.41**		
SG-C 48916	**697**	3.55	8.6	**3.66**	4.94	SG-C 48916	**698**	4.15	8.4	**4.29**	4.56	**92.29**
	**712**	5.05	8.6	**5.21**			**711**	5	8.3	**5.17**		
	**779**	5.8	8.9	**5.97**			**780**	4.1	8.5	**4.23**		
SG-C 21215	**699**	4.95	7.8	**5.14**	5.73	SG-C 21215	**700**	4.7	7.7	**4.89**	5.15	**89.82**
	**726**	5.85	8.7	**6.03**			**725**	5.4	8.3	**5.58**		
	**795**	5.8	7.9	**6.02**			**796**	4.8	8.2	**4.97**		
Navajo	**701**	4.35	8.3	**4.50**	4.20	Navajo	**702**	3.95	7.8	**4.10**	4.25	**101.34**
	**720**	4	8.3	**4.14**			**719**	4.3	8.3	**4.45**		

	**803**	3.85	8.9	**3.96**			**804**	4.1	9	**4.21**		
Sidney	**703**	4.5	8.1	**4.66**		Sidney	**704**	3.75	8.9	**3.86**		**84.71**
	**714**	5.2	8.5	**5.37**	5.13		**713**	4.5	8.4	**4.65**	4.34	
	**809**	5.2	8.8	**5.35**			**810**	4.4	8.9	**4.53**		
Arabella	**705**	4.85	7.6	**5.05**		Arabella	**706**	4.45	7.8	**4.62**		**88.95**
	**746**	5.7	7.8	**5.92**	5.53		**745**	5	8	**5.19**	4.91	
	**793**	5.4	7.9	**5.61**			**794**	4.75	7.8	**4.94**		

Supplementary Table S8. Primers used in this study.

		PCR Fragment Length (bp)			
Primer name	Sequence (5' → 3')	Resistant Genotype	Susceptible Genotype	Detection Methods	Reference
**TuYVF-K1**	TACGGTCGTGGGTAGGAGAA	185		qPCR	[11]
**TuYVR-K1**	TCGAACCTGCTCCTCTGGTA				
**R 54-1F**	GATCCGTTTGGGTCTTGGTA	344 bp	385 bp	RT-PCR	[29]
**R 54-1R**	TTGATGTGAAACGCACATTG				
**R 54-2F**	ATCGGACATTGGTCAGGTTC	437 bp	376 bp	RT-PCR	[29]
**R 54-2R**	CATACCCCACTGGTTCTTGG				
